# Macrofungal diversity in community-managed sal (*Shorea robusta*) forests in central Nepal

**DOI:** 10.1080/21501203.2015.1075232

**Published:** 2015-08-03

**Authors:** Shova Baral, Khum Bahadur Thapa-Magar, Ganesh Karki, Shiva Devkota, Bharat Babu Shrestha

**Affiliations:** a Central Department of Botany, Tribhuvan University, Kathmandu, Nepal; b School of Environment and Management Studies (SchEMS), Pokhara University, Kathmandu, Nepal; c Swiss Federal Institute for Forest, Snow and Landscape Research, WSL, Birmensdorf, Switzerland

**Keywords:** forest management, mid-hills, mycorrhiza, species richness

## Abstract

Macrofungi constitute a group of the high-value forest resources worldwide. In this paper, we report species richness and composition of the macrofungi in sal (*Shorea robusta*) forests of mid-hill central Nepal, which were managed for 4–29 years by the local communities. The sal forests were rich in macrofungi (115 species) with Polyporaceae being the largest family followed by Clavariaceae. Saprotrophic fungi were more common than mycorrhizal species. The proportion of mycorrhiza was <40% of the total macrofungi species which might have indicated the deteriorated condition of the forests before the initiation of conservation management. However, the proportion of mycorrhizal species was slightly higher in the forests managed for >10 years than in the forests managed for short period. The species richness increased with increasing canopy and litter cover. Since silvicultural activities and resource utilization often have negative impacts to macrofungal diversity, these activities need to be optimized to keep balance between forest management and biodiversity conservation.

## Introduction

Fungi are a diverse group of organisms ranging from microscopic forms to large mushrooms. Being a major group of decomposers they are essential for the survival of other organisms in the ecosystem (Hawksworth 1991). By contributing to nutrient cycle and the maintenance of ecosystems, fungi play an important role in soil formation, fertility, structure, and improvement of any habitat (Pan et al. 2008). Macrofungi are the members of ascomycetes and basidiomycetes with large, easily observed spore-bearing structures (Mueller et al. 2007) and also considered as one of the high-value non-timber forest resources (Wang and Hall 2004). Among 1021 species of macrofungi (Ascomycetes – 147 species, Basidiomycetes – 874 species) found in Nepal (Adhikari 2012), 228 species have food value (Christensen et al. 2008), while 73 species are medicinal and 65 species poisonous (Adhikari 2009).

The macrofungal species composition and diversity vary with nutrient (particularly nitrogen), moisture, forest type, disturbance, etc. (Trudell and Edmonds 2004; Christensen and Heilmann-Clausen 2009; López-Quintero et al. 2012; O’Hanlon and Harrington 2012; Pradhan et al. 2013). Climatic conditions as well as phyto-geomorphologic features affect macrofungal fructification (Brunner et al. 1992; Yang et al. 2006) and thus the chances of their collection during inventory. Forest management activities can also play a crucial role in shaping macrofungal communities since they can modify vegetation parameters and soil conditions (Wiensczyk et al. 2002).

Sal (*Shorea robusta* Gaertn.) forest, mainly found in Nepal, India, and Bhutan, has very important role in biodiversity conservation, forest economy, and people’s livelihood in the region (Gautam and Devoe 2006). While much information is available on composition and diversity of vascular plants in sal forests, ecological study of macrofungi is virtually non-existent except a few studies from West Bengal, India (Pradhan et al. 2012, 2013). In this paper, we report the impact of the duration of forest management and the stand characteristics on macrofungal species composition and richness of sal forests in mid-hill region of central Nepal. Together with similar information related to other life forms (e.g., vascular plants) the results of the present study will be helpful to assess the impact of community forestry practices on biodiversity of the forests in general.

## Materials and methods

### Research site

**Figure 1.**
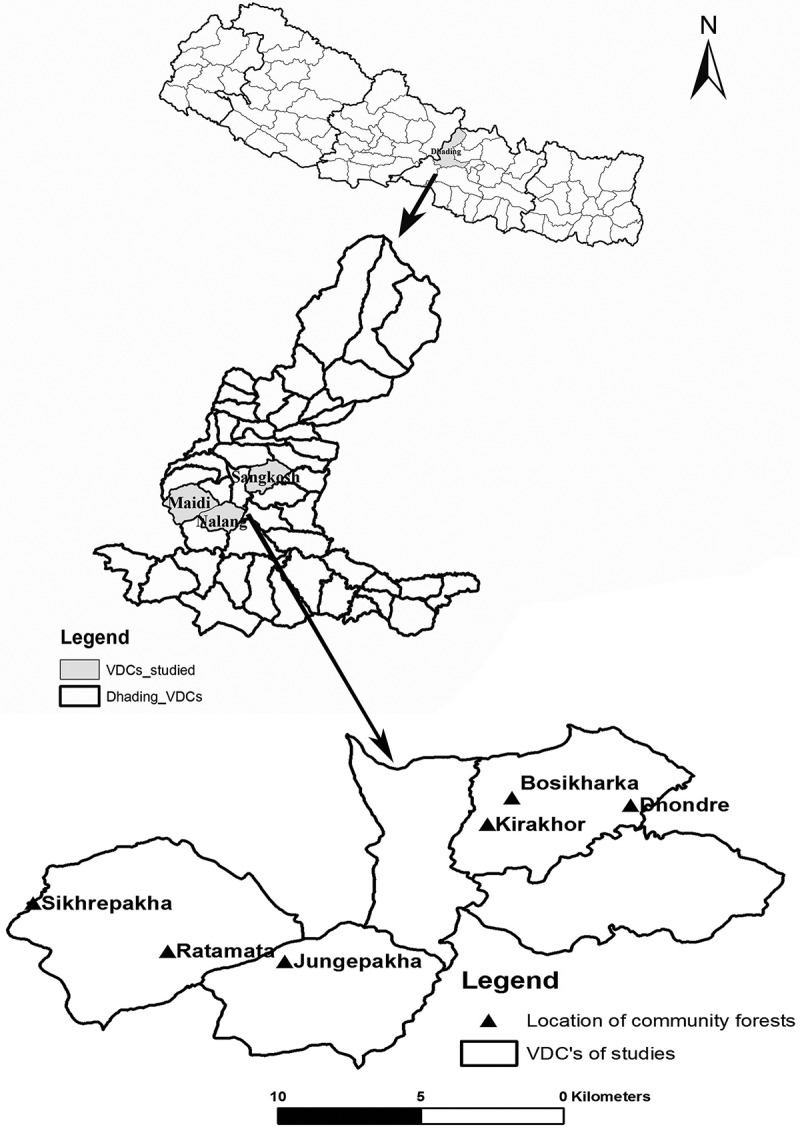
Location map of study area, showing position of Dhading district in Nepal: VDCs in district and studied forest in VDCs.

The study was carried out in mid-hill region of Dhading district in central Nepal (Figure 1). The study area has subtropical climate and receives an average 1650 mm of annual rainfall with maximum monthly rainfall during July (435 mm) (Practical Action 2009). The monthly mean temperature is maximum in June (31.54°C) and minimum in January (8.15°C). The present study was carried out in six community-managed forests (CFs) of the district which have been categorized into two groups based on management duration, that is, CFs managed for <10 years and those managed for >10 years. The CFs are the part of national forest that is handed to local Community Forest Users’ Group (CFUG) for conservation and sustainable utilization of forest resources (Bartlett 1992). All the CFs included in the present study are ‘hill sal forests’ (*sensu,* Stainton 1972) with sal as a dominant tree species and *Schima wallichii* (DC.) Korth., *Lagerstroemia parviflora* Roxb., *Cleistocalyx operculatus* (Roxb.) Merr. and Perry., etc., as associated tree species.

### Study design

The district forest office (DFO) has divided the district into eight management units (range post area) and the Neelkantha range post area was selected for study as it had the highest number (181) of CFs in the district (DFO Dhading, 2009). Of the total CFs in the area, 30 were selected randomly. In the preliminary study, during 30 May to 13 June 2010, the durations of community management of these 30 CFs were obtained from the interaction with members of the individual CFUGs. Forests dominated by species other than sal and the plantation forests were excluded from the list. Then, the remaining CFs were divided into two categories: CFs managed for <10 years which included the CFs managed for 4–6 years, and those managed for >10 years which included the CFs managed for 11–29 years. From each category, three CFs were selected randomly for sampling (Table 1). On the basis of the forest area, the number of plots to be sampled was determined so as to represent 0.12–0.5% of the forest area. Small number of sample plots in large forest such as Dhondre CF was due to steep topography; more than two-third of this forest is very steep with slope >30° which we excluded during sampling. Thirty-two plots were sampled in the CFs managed for <10 years and 19 plots in the CFs managed for >10 years. Altogether 51 plots were sampled in 6 CFs.

**Table 1.** The community-managed forests (CFs) selected for the sampling.

S.N	Name of CF	Area (ha)	Number of plots	Elevation (m asl)	Tree canopy cover (%)	Litter cover (%)	Location^a^	Management duration (years)	Category based on management duration
1	Sikrepakha	10.1	12	511	55	70	Maidi – 9	6	CF managed for <10 years
2	Kirakhor	6.2	7	906	75	80	Sankosh – 1	6	
3	Bosikharka	12.5	13	993	75	75	Sankosh – 5	4	
4	Dhondre	30.6	5	896	45	55	Sankosh – 8	11	CF managed for >10 years
5	Jungepakha	8.54	6	842	75	90	Nalang – 1	22	
6	Ratamata	18.2	8	787	80	90	Maidi – 5	29	

Note: ^a^Village development committee (VDC) area and the ward number.

### Field sampling

Field sampling was carried out from 4 to 23 August 2010, and each sample plot was visited only once. In each of the CFs, 5–13 plots (10 m × 10 m) were located by stratified random sampling method. Each CF had been divided into a variable number (2–5) of blocks by the CFUGs for management; these blocks were considered as ‘strata.’ Each block in the map was divided into a large number of plots and a unique number was assigned to each of them. Then, the desired number of plots was selected randomly. With the help of local people, who could read the map, the location of selected plots was identified in the field. In each plot, all species of macrofungi were collected and photographed. Geographic location and slope were recorded using global positioning system (GPS) and clinometer. Litter cover (%) and tree canopy (%) were estimated visually from the center of each plot. Samples of macrofungi were dried and stored in wax-coated paper bags. Identification manuals (e.g., Adhikari 2000; Fries 1838; Bakshi 1971; Dickinson and Lucas 1979; Phillips 1981; Thind and Sharma 1983; Pacioni 1985; Imazeki et al. 1988; Kumar et al. 1990) were used for identification.

Soil sample was collected from each plot to determine organic carbon. Each plot was divided into four subplots (5 m × 5 m) and soil samples from 15 cm depth were taken from the center of each subplot; these four samples were mixed thoroughly and approximately 200 g soil was finally taken in a plastic bag for laboratory analysis. After a week of air drying in shade, soil organic matter was determined by Walkley Black method (Zobel et al. 1987).

### Data analysis

Similarity in species composition between the two categories of forests was estimated as Jaccard’s similarity index (Zobel et al. 1987). Frequency (%) of species in each forest category was calculated as follows: number of plots with a particular species × 100/total number of plots sampled. Species richness (mean number of species per plot) of macrofungi in the two categories of CFs was compared by independent sample *t*-test. The *χ*^2^ test was used to test if there is any relation between management duration of the CFs (<10 years vs. >10 years) and species composition based on trophic groups (mycorrhizal vs. saprotrophic fungi). Only a few species were parasitic, therefore this trophic group was excluded from the analysis. Regression was used to estimate the effects of tree canopy, litter cover, and soil organic carbon on macrofungi species richness. For regression analyses, plot-wise values of both response (i.e., macrofungal species richness) and explanatory variables (i.e., tree canopy, litter cover, and soil organic matter) were pooled together. Statistical Package for Social Science (SPSS, version 11.0) was used for statistical analyses.

## Results

### Species richness and composition

**Figure 2.**
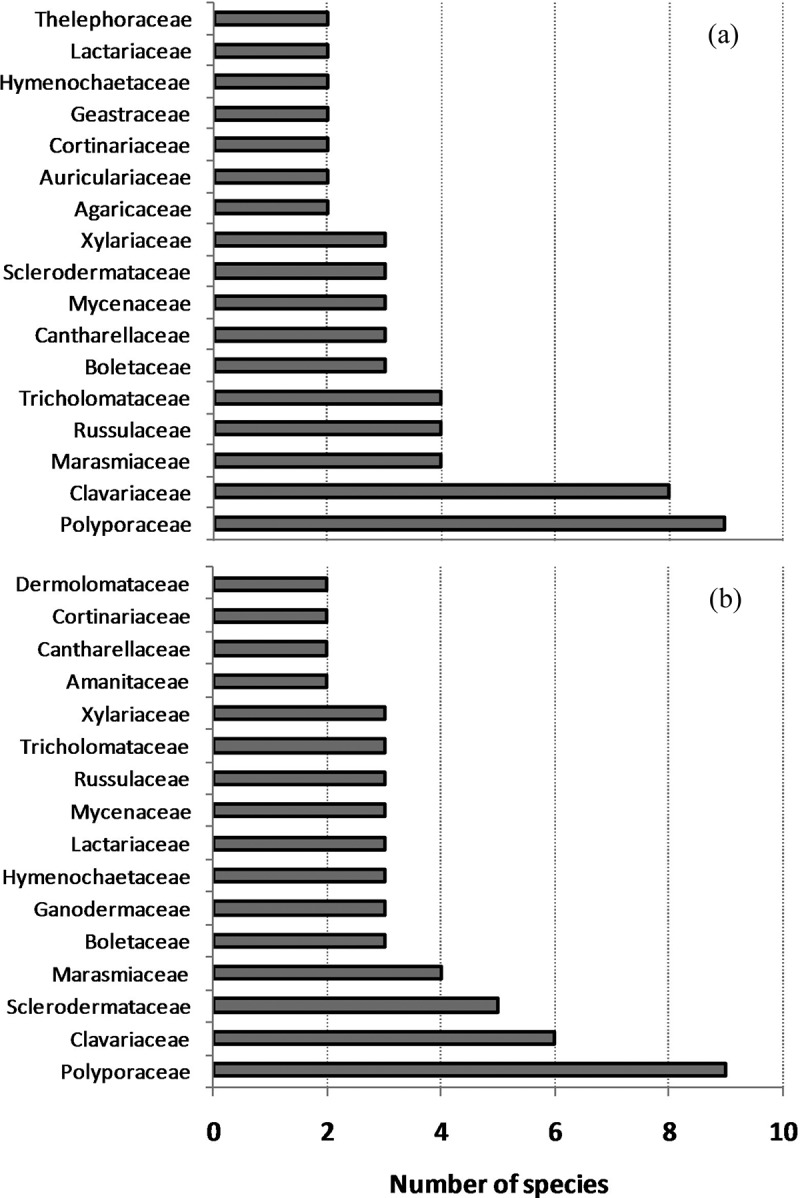
Number of species belonging to different families of macrofungi in the forests managed for (a) <10 years and (b) >10 years. All other families were represented by single species and their list can be found in Supplementary Table 2.

We recorded 84 species of macrofungi in sal forests managed for <10 years and 73 species in the forests managed for >10 years (Table 2, Supplementary Table 1). In both types of forests, the number of species belonging to different trophic groups were in the order of saprotrophic > mycorrhizal > parasitic species. Polyporaceae was the largest family followed by Clavariaceae in both categories of forests (Figure 2). Seventeen families in forests managed for <10 years and 16 families in forests managed for >10 years were represented by >1 species. Eighteen and 12 families were represented by one species in forests managed for <10 years and >10 years, respectively (Supplementary Table 2). Species richness of macrofungi in forests managed for short duration of time (<10 years) was nine, while it was eight in the forests managed for long duration (>10 years); these values were not significantly different (independent sample *t*-test, *p* > 0.05).

**Table 2.** Species richness of the macrofungi in two categories of forests.

Attributes	Management duration	
	<10 years	>10 years
Total number of species	84	73
Number of species not identified	8	5
Number of families	35	28
Saprotrophic species^a^	50 (66%)	38 (56%)
Mycorrhizal species^a^	23 (30%)	24 (35%)
Parasitic species^a^	3 (4%)	6 (9%)
Species richness (#species/plot)	8.94 ± 3.15	8.63 ± 2.52

Note: ^a^Percentage was calculated based on the total number of known species.

Jaccard’s similarity index between macrofungi in the CFs of two different management durations was 34%. Out of the 103 identified taxa, 42 were common to the forests with different management durations. This shows that 42 species were present only in the CFs managed for <10 years and 31 species in CFs managed for >10 years.

Frequency of macrofungal species ranged from 3% to 44% in the CFs managed for <10 years, and from 5% to 42% in the CFs managed for >10 years (Supplementary Table 1). Most of the species having high frequency were found in both forest categories. *Coltricia cinnamomea, Cantharellus leucocomus, Laccaria laccata, Clavaria vermicularis*, and *Russula aurora* were the common and frequent species present in both categories of forests (Table 3).

**Table 3.** Ten most frequent species in two different categories of community-managed forest.

Forest managed for <10 years			Forest managed for >10 years		
SN	Name of the species	Frequency (%)	SN	Name of species	Frequency (%)
1.	*Coltricia cinnamomea* (Jacq.: Fr.) Karst.	44	1.	*Coltricia cinnamomea* (Jacq.: Fr.) Karst.	42
2.	*Russula aurora* (Krombh)	41	2.	*Cantharellus leucocomus* Bigelow	32
3.	*Cantharellus leucocomus* Bigelow	41	3.	*Laccaria laccata* (Scop.: Fr.) Cooke	32
4.	*Scleroderma cepa* (Pers.) Fr.	31	4.	*Lactarius volemus* (Fr.) Fr.	26
5.	*Clavaria vermicularis* Swartz: Fr.	31	5.	*Marasmius siccus* (Schwein.) Fr.	26
6.	*Campanella caesia* Romagn	31	6.	*Clavaria vermicularis* Swartz: Fr.	26
7.	*Collybia cirrhata* (Sesu Cooke)	28	7.	*Clavaria acuta* Sch.: Fr.	26
8.	*Laccaria laccata* (Scop.: Fr.) Cooke	25	8.	*Clavariadelphus pistillaris* (L.) Donk	21
9.	*Lactarius volemus* (Fr.) Fr.	25	9.	*Russula aurora* (Krombh)	21
10.	*Cantharellus* sp.	25	10.	*Scleroderma bovista* Fr.	21

Any shift in trophic groups (mycorrhizal vs. saprotrophic fungi) with management duration of the forest was tested using *χ*^2^ test. The calculated value of *χ*^2^ was smaller than the tabulated value of *χ*^2^ at *p* = 0.05 indicating that there was no relation between management duration of the forests and the species of different trophic behavior.

### Effect of environmental variables on macrofungal species richness

**Figure 3.**
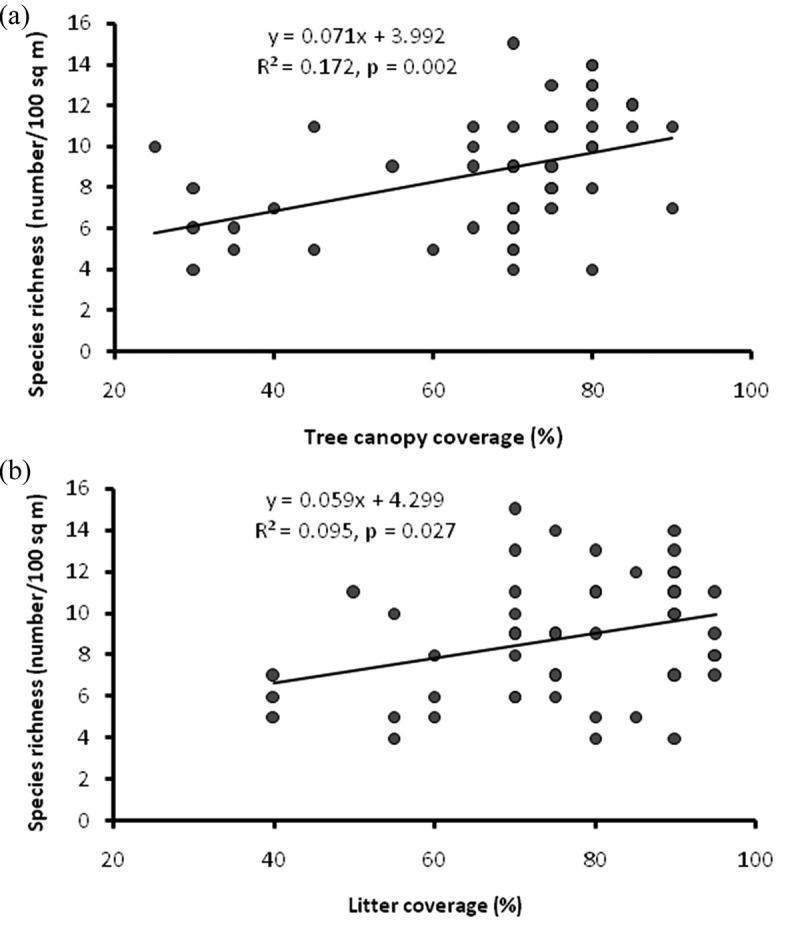
Variation in species richness of macrofungi with tree canopy (a) and litter cover (b).

In the studied CFs, tree canopy, litter cover, and soil organic matter varied 25–90%, 40–95%, and 0.77–3.44%, respectively. Among the three environmental variables considered, tree canopy and litter cover had significant positive impact on species richness of macrofungi in the studied forests (Figure 3). We could not establish any significant relationship between soil organic carbon and species richness of macrofungi (linear regression, *p* > 0.05).

## Discussion

### Macrofungal species richness and composition

The community-managed sal forests of the mid-hill region of Nepal seem to support a large number of macrofungi species. As compared with the reported 60 species of macrofungi in 90 (20 m × 20 m) plots in sal forests of lateritic region of West Bengal, India (Pradhan et al. 2012), occurrence of >100 species in 51 (10 m × 10 m) plots indicates the richness of macrofungi in the present studied forests. Although all the CFs studied lies in the same climatic region, they were isolated patches at different stages of forest development. Therefore, relatively high number of species might be related to high microhabitat variation.

Proportion of mycorrhizal fungi is often considered as the indicator of forest health and it is high (often >50% of total macrofungi) in healthy, productive, and undisturbed forests (Arnolds 1988). In some old-grown forests, the number of mycorrhizal fungi species was 2.4 times higher than the saprotrophic fungi (Richard et al. 2004). The forests having high proportion of mycorrhizal fungi are also considered better conserved than the forests having low proportion (Ortega and Lorite 2007). Furthermore, silviculture-related disturbances in forests also reduce the proportion of mycorrhizal fungi and corresponding increase in saprotrophic species (Kropp and Albee 1996; Hartmann et al. 2014). In CFs managed both for short and long duration, mycorrhizal fungi contributed <40% of the total macrofungi, which has been considered ‘acute’ in terms of forest deterioration by Fellner and Pešková (1995). This might have reflected the deteriorated condition of the CFs before conservation management was initiated by the local communities. Irrespective of the different management durations, all the CFs included in the present study were highly degraded with sparse shrubby vegetation and a few isolated trees before the management was initiated by the local communities. In terms of tree stocking, the management by CFUG has had positive impact on these CFs (Thapa-Magar and Shrestha 2015). Slightly higher proportion of mycorrhizal fungi in the CFs managed for >10 years (35%) than in the CFs managed for <10 years (30%) might be the result of improvement in forest health after conservation. Change in species composition of macrofungi, as a part of fungal succession (Frankland 1998), is also apparent from the very low value of similarity index between two categories of forests, though there was no difference in plot-wise species richness. However, the concept of acute deterioration at early stages and subsequent improvement after conservation management cannot be generalized to community-managed sal forests of Nepal without having data from the landscape level.

### Variation of macrofungal species richness with environmental variables

Fungal diversity is closely related with forest structure and composition (Richard et al. 2004). In the present studied forests, species richness increased with increasing tree canopy cover (Figure 3(a)). This coincides with the observation made by Dighton et al. (1986) that the greatest species diversity of fungus seems to occur where there is canopy closure. Sysouphanthong et al. (2010) also reported higher macrofungal diversity in forests having higher canopy closure. In forest stands with medium density (and canopy cover), the mycorrhizal species produced twice as many fruit bodies as in the stands with low density, whereas saprotrophic species did not differ significantly (Ayer et al. 2006). Thinning of trees caused a decline in fruit-body production of mushroom, but this effect varied greatly according to the season and to the pattern and level of thinning (Luoma et al. 2004). Therefore, thinning and pruning, which are the common silvicultural activities in the CFs of Nepal (Shrestha et al. 2010), might have also some effects on the composition and diversity of macrofungi.

Litter is an important component of all ecosystems and constitutes the major source of organic matter. The removal of litter affects fungal growth and diversity (Eaton et al. 2004; Sayer 2005). Species richness of macrofungi therefore increased with increasing litter cover (Figure 3(b)). When the forest floor is covered with layers of well-decomposed leaves, saprotrophic fungi are favored by this organic resource which maintains temperature and moisture (Fernández-Toirán et al. 2006). Although the abundance of macrofungal species is closely correlated with soil organic matter and other soil parameters (Zamora-Martinez and DePascual-Pola 1995; Engola et al. 2007), we could not establish any relationship between soil organic matter and macrofungi species richness. It is likely that: (1) one-growing season data are inadequate to measure the fungal diversity (Straatsma and Krisai-Greilhuber 2003), and (2) the effect of soil organic carbon on species richness might have been overridden by other factors such as disturbances and succession.

## Conclusion

The community-managed sal forests of mid-hill region of central Nepal were rich in macrofungal species. The proportion of mycorrhizal species was slightly higher in the CFs managed for >10 years than in the CFs managed for shorter period. The species richness increased with increasing canopy and litter cover. Since silvicultural activities and resource utilization often have negative impacts to macrofungal diversity, these activities need to be optimized to keep balance between forest management and biodiversity conservation.

## Disclosure statement

No potential conflict of interest was reported by the authors.

## Supplemental data

Supplemental data for this article can be accessed at http://dx.doi.10.1080/21501203.2015.1075232.

Supplementary_files.zip
