# Supplementary material for: Macrofungal diversity in community-managed sal (Shorea robusta) forests in central Nepal
Source: Mycology. 2015 Aug 3;6(3-4):151–7. doi: 10.1080/21501203.2015.1075232 (PMC6106073; doi:10.1080/21501203.2015.1075232)
Supplement: Supplementary_files.zip [file TMYC_A_1075232_SM3417.zip › Supplementary files/Supplementary Table 1.docx]

**Supplementary Table 1. List of Macrofungi in the community forest managed for <10 years and > 10 years**

| **S.N** | **Scientific name** | **Family** | **Trophic group** | **Frequency** | |
| --- | --- | --- | --- | --- | --- |
|  |  |  |  | **< 10** | **> 10** |
| 1 | *Amanita fulva* (Schaeff. : Fr.) Fr*.* | Amanitaceae | Mycorrhizae | - | 5.26 |
| 2 | *Amanita pantherina* (DC.: Fr.) Kromb. | Amanitaceae | Mycorrhizae | - | 5.26 |
| 3 | *Anthracobia macrocystis* (Cke.) Bound. | Pyronemataceae | Saprotrophic | 12.5 | - |
| 4 | *Auricularia auricula-judae* (Bull.: Fr.) Wettst. | Auriculariaceae | Parasitic | 3.13 | - |
| 5 | *Bisporella citrina* (Batsch.: Fr.) Korf. & Carp. | Helotiaceae | Saprotrophic | 15.63 | - |
| 6 | *Bolete* sp. | Boletaceae | Mycorrhizae | - | 5.26 |
| 7 | *Bolete* sp. 3 | Boletaceae | Mycorrhizae | - | 10.53 |
| 8 | *Bolete* sp*.*1 | Boletaceae | Mycorrhizae | 12.5 | - |
| 9 | *Bolete* sp.4 | Boletaceae | Mycorrhizae | - | 5.26 |
| 10 | *Bolete* sp.2 | Boletaceae | Mycorrhizae | 3.13 | - |
| 11 | *Boletellus* sp. | Boletaceae | Mycorrhizae | 3.13 | - |
| 12 | *Calocera cornea* (Batsch.: Fr.) Korf. & Carp*.* | Dacrymycetaceae | Saprotrophic | 3.13 | - |
| 13 | *Campanella caesia.* Romagn | Marasmiaceae | Saprotrophic | 31.25 | 15.79 |
| 14 | *Cantharellus leucocomus* Bigelow. | Cantharellaceae | Mycorrhizae | 40.63 | 31.58 |
| 15 | *Cantharellus* sp. | Cantharellaceae | Mycorrhizae | 25 | 15.79 |
| 16 | *Clavaria acuta* Sch.: Fr. | Clavariaceae | Saprotrophic | - | 5.26 |
| 17 | *Clavaria fragilis* Fr. | Clavariaceae | Saprotrophic | 9.38 | - |
| 18 | *Clavaria rosea* Fr. | Clavariaceae | Saprotrophic | 3.13 | - |
| 19 | *Clavaria* sp. | Clavariaceae | Saprotrophic | - | 10.53 |
| 20 | *Clavaria* sp*. 1* | Clavariaceae | Saprotrophic | 12.5 | - |
| 21 | *Clavaria* sp. 2 | Clavariaceae | Saprotrophic | 3.13 | - |
| 22 | *Clavaria vermicularis* Swartz: Fr. | Clavariaceae | Saprotrophic | 31.25 | 15.79 |
| 23 | *Clavariadelphus pistillaris* (L.) Donk | Clavariaceae | Saprotrophic | 6.25 | 21.05 |
| 24 | *Clavulina* sp. 2 | Clavariaceae | Saprotrophic | 6.25 | - |
| 25 | *Clavulina* sp. 2 | Clavariaceae | Saprotrophic | 25 | 26.32 |
| 26 | *Clavulina* sp.3 | Clavariaceae | Saprotrophic | - | 5.26 |
| 27 | *Clitocybe* sp. | Tricholomataceae | Saprotrophic | 9.38 | - |
| 28 | *Collybia cirrhata* (Sesu Cooke) | Tricholomataceae | Saprotrophic | 28.13 | 15.79 |
| 29 | *Coltricia cinnamomea* Jacq.: Fr.) Karst. | Hymenochaetaceae | Saprotrophic | 43.75 | 42.11 |
| 30 | *Coprinus disseminatus* (Pers.:Fr) Gray | Hymenochaetaceae | Saprotrophic | 6.25 | 10.53 |
| 31 | *Cortinarius* sp. 1 | Cortinariaceae | Mycorrhizae | 3.13 | 5.26 |
| 32 | *Cudonia* sp. | Cudoniaceae | Saprotrophic | 3.13 | 5.26 |
| 33 | *Dacrymyces stillatus* Nees. | Dacrymycetaceae | Parasitic | - | 5.26 |
| 34 | *Daedaleopsis* sp. | Polyporaceae | Saprotrophic | - | 5.26 |
| 35 | *Daldinia concentrica* (Bull.: Fr.) Ces. & De | Xylariaceae | Saprotrophic | 9.38 | 5.26 |
| 36 | *Exidia glandulosa* (Bull.: Fr.) Wettst*.* | Auriculariaceae | Parasitic | 3.13 | - |
| 37 | *Flamulina* sp. | Dermolomataceae | Saprotrophic | 18.75 | 15.79 |
| 38 | *Ganoderma lucidum* (Curtis) P. Karst*.* | Ganodermaceae | Parasitic | - | 5.26 |
| 39 | *Ganoderma resinaceum* Boud*.* | Ganodermaceae | Parasitic | - | 5.26 |
| 40 | *Ganoderma* sp. | Ganodermaceae | Parasitic | - | 5.26 |
| 41 | *Geastrum* sp. | Geasterace | Saprotrophic | - | 5.26 |
| 42 | *Geastrum* sp. 2 | Geastraceae | Saprotrophic | 3.13 | - |
| 43 | *Geastrum* sp*.*1 | Geastraceae | Saprotrophic | 15.63 | - |
| 44 | *Helvella* sp*.* | Helvellaceae | Saprotrophic | 3.13 | - |
| 45 | *Hydnum repandum* L.: Fr*.* | Hydnaceae | Mycorrhizae | 15.63 | 10.53 |
| 46 | *Hygrocybe lanecovensis* A. M. Young | Hygrophoraceae | Mycorrhizae | 9.38 | - |
| 47 | *Inocybe* sp*.* | Inocybaceae | Mycorrhizae | 6.25 | 10.53 |
| 48 | *Inonotus* sp. | Hymenochaetaceae | Parasitic | - | 5.26 |
| 49 | *Irpex* sp. | Meruliaceae | Saprotrophic | 3.13 | - |
| 50 | *Irpex* sp.2 | Meruliaceae | Saprotrophic | - | 5.26 |
| 51 | *Kobayasia nipponica* Imai & Kawam | Sclerodermataceae | Mycorrhizae | - | 15.79 |
| 52 | *Laccaria amesthesia* (Hunds.) Cooke | Tricholomataceae | Mycorrhizae | - | 5.26 |
| 53 | *Laccaria* *laccata* (Scop. : Fr.) Cooke | Tricholomataceae | Mycorrhizae | 25 | 31.58 |
| 54 | *Lactarius indigo* (Schew.) Fr*.* | Lactariaceae | Mycorrhizae | - | 5.26 |
| 55 | *Lactarius* sp. | Lactariaceae | Mycorrhizae | 9.38 | 5.26 |
| 56 | *Lactarius volemus* (Fr.) Fr*.* | Lactariaceae | Mycorrhizae | 25 | 26.32 |
| 57 | *Lentinus* sp. | Pleurotaceae | Saprotrophic | 3.13 | - |
| 58 | *Leotia lubrica* (Scop.) Pers*.* | Leotiaceae | Saprotrophic | - | 5.26 |
| 59 | *Lepiota cristata* (Alb. & Schw.) | Agaricaceae | Saprotrophic | 12.5 | 10.53 |
| 60 | *Lepiota* sp*.* | Agaricaceae | Saprotrophic | 3.13 | - |
| 61 | *Marasmius androsaceus* (L. ex Fr.) | Marasmiaceae | Saprotrophic | 6.25 | 5.26 |
| 62 | *Marasmius candidus* (Bolt.) Singer | Marasmiaceae | Saprotrophic | 18.75 | 26.32 |
| 63 | *Marasmius siccus* (Schwein.) Fr | Marasmiaceae | Saprotrophic | 15.63 | 26.32 |
| 64 | *Microporus* sp. | Polyporaceae | Saprotrophic | 9.38 | - |
| 65 | *Microporus xanthopus* (Fr.) Kuntze*.* | Polyporaceae | Saprotrophic | 12.5 | 15.79 |
| 66 | *Mycena galericulata* (Scop.: Fr) S.F. Gray. | Mycenaceae | Saprotrophic | 3.13 | - |
| 67 | *Mycena galopus* (Pers.) P. Kumm. | Mycenaceae | Saprotrophic | 6.25 | 15.79 |
| 68 | *Mycena pura* (Pers.) P. Kumm. | Mycenaceae | Saprotrophic | - | 15.79 |
| 69 | *Mycena* sp*.* | Mycenaceae | Saprotrophic | 3.13 | 15.79 |
| 70 | *Mycorraphium adjustum* (Schw.)Gees | Cantharellaceae | Saprotrophic | 3.13 | - |
| 71 | *Oudemasiella* sp. | Dermolomataceae | Saprotrophic | - | 5.26 |
| 72 | *Paneolus* sp*.* | Bolbitiaceae | Saprotrophic | 3.13 | - |
| 73 | *Peziza* sp*.* | Pezizaceae | Saprotrophic | 3.13 | - |
| 74 | *Pholiota terrestris* Overh. | Cortinariaceae | Saprotrophic | 6.25 | 5.26 |
| 75 | *Polypore* sp. 7 | Polyporaceae | Saprotrophic | - | 5.26 |
| 76 | *Polypore* sp. 8 | Polyporaceae | Saprotrophic | - | 5.26 |
| 77 | *Polypore* sp. 1 | Polyporaceae | Saprotrophic | 3.13 | - |
| 78 | *Polypore* sp*.* 2 | Polyporaceae | Saprotrophic | 6.25 | - |
| 79 | *Polypore* sp. 3 | Polyporaceae | Saprotrophic | 3.13 | 10.53 |
| 80 | *Polypore* sp*.* 4 | Polyporaceae | Saprotrophic | 3.13 | - |
| 81 | *Polypore* sp. 5 | Polyporaceae | Saprotrophic | 6.25 | - |
| 82 | *Polyporellus brumalis* (Pers.: Fr.) Karst. | Polyporaceae | Saprotrophic | 6.25 | 5.26 |
| 83 | *Polyporus* sp*.* | Polyporaceae | Saprotrophic | - | 5.26 |
| 84 | *Pycnoporus cinnabarinus* (Jacq.: Fr.) Karst. | Polyporaceae | Saprotrophic | 12.5 | 5.26 |
| 85 | *Ramaria flaccida* (Fr.: Fr.) Bourdot | Ramariaceae | Saprotrophic | 6.25 | - |
| 86 | *Rhizopogon* sp. | Sclerodermataceae | Mycorrhizae | 3.13 | 5.26 |
| 87 | *Russula aurora* (Krombh) Bres. | Russulaceae | Mycorrhizae | 40.63 | 21.05 |
| 88 | *Russula delica* Fr. | Russulaceae | Mycorrhizae | 15.63 | 10.53 |
| 89 | *Russula flavida* Frost*.* | Russulaceae | Mycorrhizae | 3.13 | 15.79 |
| 90 | *Russula* sp*.* | Russulaceae | Mycorrhizae | 9.37 | - |
| 91 | *Schizophyllum commune* Fr: Fr. | Schizophyllaceae | Saprotrophic | 6.25 | 5.26 |
| 92 | *Scleroderma bovista* Fr. | Sclerodermataceae | Mycorrhizae | 12.5 | 21.05 |
| 93 | *Scleroderma cepa* Pers. | Sclerodermataceae | Mycorrhizae | 31.25 | 15.79 |
| 94 | *Scleroderma* sp. | Sclerodermataceae | Mycorrhizae | - | 5.26 |
| 95 | *Thelophora palmata* Fr.: Fr | Thelophoraceae | Mycorrhizae | 3.13 | - |
| 96 | *Thelophora* sp. | Thelophoraceae | Mycorrhizae | 6.25 | 5.26 |
| 97 | *Trametes versicolor* (L.: Fr.) Llyod. | Polyporaceae | Saprotrophic | - | 10.53 |
| 98 | *Tremella mesenterica* Ritz.: Fr*.* | Tremellaceae | Parasitic | 3.13 | 5.26 |
| 99 | *Termitomyces* sp*.* | Lyophyllaceae | Mycorrhizae | 12.5 | - |
| 100 | *Trichoglossum hirsutum* (Pers.: Fr.) Bound. | Geoglossaceae | Saprotrophic | 12.5 | 5.26 |
| 101 | *Tricholoma* sp. | Tricholomataceae | Mycorrhizae | 3.13 | - |
| 102 | *Xylaria filiformis* (Alb. & Schwein) Fr. | Xylariaceae | Saprotrophic | 6.25 | 15.79 |
| 103 | *Xylaria hypoxylon* (L.) Grev. | Xylariaceae | Saprotrophic | 3.13 | 10.53 |

Note: There were twelve specimens which could not be identified even to family level and thus there could not be listed here.
