# Supplementary material for: Macrofungal diversity in community-managed sal (Shorea robusta) forests in central Nepal
Source: Mycology. 2015 Aug 3;6(3-4):151–7. doi: 10.1080/21501203.2015.1075232 (PMC6106073; doi:10.1080/21501203.2015.1075232)
Supplement: Supplementary_files.zip [file TMYC_A_1075232_SM3417.zip › Supplementary files/Supplementary table 2.docx]

**Supplementary table 2. Number of species belonging to different families of macrofungi.**

| **Family** | **Management duration (Years)** | |
| --- | --- | --- |
|  | **<10 years** | **>10 years** |
| Agaricaceae | 2 | 1 |
| Amanitaceae | - | 2 |
| Auriculariaceae | 2 | - |
| Bolbitiaceae | 1 | - |
| Boletaceae | 3 | 3 |
| Cantharellaceae | 3 | 2 |
| Clavariaceae | 8 | 6 |
| Cortinariaceae | 2 | 2 |
| Cudoniaceae | 1 | 1 |
| Dacrymycetaceae | 1 | 1 |
| Dermolomataceae | 1 | 2 |
| Ganodermaceae | - | 3 |
| Geastraceae | 2 | 1 |
| Geoglossaceae | 1 | 1 |
| Helotiaceae | 1 | - |
| Helvellaceae | 1 | - |
| Hydnaceae | 1 | 1 |
| Hygrophoraceae | 1 | - |
| Hymenochaetaceae | 2 | 3 |
| Inocybaceae | 1 | 1 |
| Lactariaceae | 2 | 3 |
| Leotiaceae | - | 1 |
| Lyophyllaceae | 1 | - |
| Marasmiaceae | 4 | 4 |
| Meruliaceae | 1 | 1 |
| Mycenaceae | 3 | 3 |
| Pezizaceae | 1 | - |
| Pleurotaceae | 1 | - |
| Polyporaceae | 9 | 9 |
| Pyronemataceae | 1 | - |
| Ramariaceae | 1 | - |
| Russulaceae | 4 | 3 |
| Schizophyllaceae | 1 | 1 |
| Sclerodermataceae | 3 | 5 |
| Thelephoraceae | 2 | 1 |
| Tremellaceae | 1 | 1 |
| Tricholomataceae | 4 | 3 |
| Xylariaceae | 3 | 3 |
